# Fabrication of Color Glass by Pearlescent Pigments and Dissolved EVA Film

**DOI:** 10.3390/ma15165570

**Published:** 2022-08-13

**Authors:** Seongmin Lim, Hyeon-Sik Ahn, Akpeko Gasonoo, Jae-Hyun Lee, Yoonseuk Choi

**Affiliations:** 1 Department of Electronic Engineering, Hanbat National University, Daejeon 34158, Korea; 2 Department of Chemistry, University of Calgary, 2500 University Drive N.W., Calgary, AB T2N 1N4, Canada; 3 Department of Creative Convergence Engineering, Hanbat National University, Daejeon 34158, Korea

**Keywords:** BIPV, pearlescent pigment, ethylene vinyl acetate (EVA), solution process

## Abstract

In this paper, we propose a single-layer thin-film color glass manufacturing process for building-integrated photovoltaics (BIPV) with excellent aesthetics and high transmittance, through a solution process using pearlescent pigments. As a matrix for the color solution, ethylene vinyl acetate (EVA), which serves as an encapsulant and adhesive for the photovoltaic module (PV), was dissolved and used as a matrix for the color solution. The color glass produced is excellent in securing the aesthetics of buildings, has a high transmittance of 90% or more, outputs a maximum solar power generation efficiency of 91% from a solar cell, and can minimize the deterioration of power generation efficiency. In addition, the characteristics do not change over time, so it is suitable as color glass for BIPV. Through this study, the solution-based color glass manufacturing process for BIPV using dissolved EVA as a matrix forms a single-layer thin film with good color extensions. The choice of EVA as a matrix makes it possible for color glass to be easily attached to a solar panel using a heat press method. This proposed technique makes it easier and simpler to manufacture color glass as compared to the physical vapor deposition process. The adoption of this solution process technique to fabricate pearlescent pigment-based color glass can effectively reduce the time and cost of the process, so it is expected to be applied to the low-cost BIPV market with excellent aesthetics and high transmittance.

## 1. Introduction

Recently, with the development of technology and the improvement of living standards, energy consumption is rapidly increasing, and solving environmental problems such as global warming and sea levels rising is a global concern [1]. Recently, various eco-friendly renewable energy generation methods have been studied to replace fossil fuels, and to limit carbon emissions and decrease petroleum resources. Among various renewable energies, solar energy has proven to be an excellent candidate since it is clean and eco-friendly [2]. A building-integrated photovoltaic (BIPV) system, which can produce electric energy by using solar energy, is a representative photovoltaic (PV) power generation system [3,4]. As a power generation system that integrates power generation panels, it absorbs sunlight from the building itself and generates electric energy required for the building [5,6]. Through this self-generation mechanism, it is possible to efficiently reduce the dependence on external energy supply, thereby effectively lowering carbon emissions and resource consumption [7]. Most of the existing solar power generation systems are installed on a large area. However, the construction of such photovoltaic power generation systems shows the area limits power generation efficiency. Hence a photovoltaic power generation system that can sufficiently supply or help the energy used in the building is required. Since the BIPV system integrates the solar power panel with the exterior wall of the building, rather than constructing a photovoltaic power panel on the roof of a building or a large outdoor area such as the conventional photovoltaic system, high power generation efficiency can be obtained [8,9,10]. As such, PV applications have shifted from small PV cells to large PV systems. To apply PV technology to buildings, PV modules have been incorporated into new buildings as façades or roofing materials, or have been increasingly integrated into existing buildings by means of a retrofit [11,12]. In BIPV, photovoltaic panels are used as exterior materials for buildings. Recently, when photovoltaic panels have been applied to the exterior walls of buildings, the overall aesthetics of the buildings has been affected, and this has therefore become another area to be investigated [13,14]. It is necessary not to only secure the power generation efficiency and performance of photovoltaic panels, but also the aesthetics of the buildings. A general photovoltaic panel is composed of black or dark blue monochromatic silicon PVs in series. When such a monochromatic solar power panel is directly applied to the exterior wall of a building, the choice of color for the exterior wall of the building is limited and becomes a factor that does not enhance the aesthetics of the building. However, if color glass is employed, it is possible to prevent deterioration of the aesthetics of the building due to the photovoltaic panel. A BIPV system is a power generation system with special conditions applied to buildings, unlike general photovoltaic power generation systems, and various factors must be considered. In the BIPV system, the goal is not to only maximize the efficiency and performance of photovoltaic power generation, but also to improve the flexibility of various designs and to create outstanding aesthetic values, which requires careful consideration of the aesthetics of the building [15,16,17]. A representative method for manufacturing color glass for BIPV is in the form of a multi-layered metal oxide thin-film structure with a suitable refractive index deposited using physical vapor deposition methods such as high-frequency sputtering and thermal evaporation [18,19]. The physical vapor deposition process is a method of laminating a metal oxide with different refractive indices in a multi-layer structure and is widely used in fabricating color glass for BIPV systems. The physical vapor deposition process controls the metal oxide thickness, transmittance, and reflection spectra, etc. Generally, the deposition of the multi-layer requires a complex process in a high temperature or vacuum environment. The multi-layered metal oxide formation using the physical vapor deposition process is highly dependent on the thickness of the thin film, so even a slight difference in thickness can affect the optical properties and color realization of the color glass [20,21,22]. Many studies have been carried out for the production of color glass, such as the use of nanoparticles [23] and metal oxides for the production of BIPV color glass [24]. Color glass implemented with nanoparticles showed about 80% transmittance in the visible light range [23]. However, the color glass manufacturing process using nanoparticles is complicated to synthesize and coat. Moreover, the use of optical adhesives such as Norland optical adhesive (NOA) increases the material cost. Other investigations reported that color glass can be manufactured using two metal oxides with different refractive indices [24]. Consequently, color glass was produced by RF sputtering. The color films exhibit low transmittances of up to 65% depending on the deposition thickness of the metal oxide used. Both color glass manufacturing processes using nanoparticles and metal oxides adopt ethylene vinyl acetate (EVA) film to laminate with solar cells. EVA is essential for laminating solar panels and glass substrates in conventional color glass structures for BIPV systems, and it is an excellent adhesive that can passivate against external factors such as moisture and dust. It also has advantages such as high transmittance, UV resistance, and weather resistance [25], implementing the color with a color solution coating and not using additional adhesives to simplify the color realization and adhesion process using EVA, which is the existing BIPV color glass manufacturing method [26]. The transmittance of color glass made using optical adhesive is 85~90%, which is similar to the color glass proposed in this paper. In the case of the color glass using NOA as a matrix, NOA is used between the back sheet and the solar cell in the lamination process performed to make the BIPV module [26]. However, NOA requires strong UV radiation to be cured. The back sheet and the solar cell could limit the transmittance to UV radiation, thereby limiting the penetration of UV radiation to cure the NOA laminate. In addition, since the viscosity of NOA is very high, it is challenging to apply large area coatings, as it should be laminated to the solar cell immediately after being coated. On the other hand, since EVA uses a basic thermocompression bonding process, BIPV modules can be made in a relatively easy way. As a result, the process of using color solutions effectively reduces the time and cost of producing color glass and laminating it to PV panels. In addition, the physical vapor deposition process for manufacturing color glass is difficult, complex, and expensive, especially when the manufacturing environment requires a high vacuum and temperature. Therefore, there is a need for an easy and simple manufacturing process for color glass for BIPV systems that does not limit solar power generation efficiency due to film thickness dependence and low transmittance.

In this paper, we propose a color glass fabrication process for BIPV by a thin single-layer film using pearlescent pigments via a solution process method. Pearlescent pigment are pigments with a plate-like crystal structure, with sizes up to a few tens of microns. Color glass is made using pearlescent pigments mixed in dissolved EVA. EVA acts as a matrix and laminating adhesive, simplifying the overall structure, manufacturing, and lamination process. Additionally, the color implementation using a solution is faster and simpler than the multi-layer metal-oxide-based color glass manufacturing technique. This advantage leads to an effective reduction in the time and cost of the color glass manufacturing process. We demonstrate six examples of color glass manufactured through this solution process. The suitability of color glass for BIPV systems was investigated through an optical characteristic analysis. Each color glass example shows a transmittance of 90% or more, thereby minimizing the deterioration in solar power generation efficiency. In addition, it was possible to measure the solar power generation efficiency by laminating the tinted glass on the solar panel and obtaining a solar power generation efficiency of up to 91%. In addition, the transmittance and solar efficiency of the color glass over time were investigated. This solution process for manufacturing high-transmittance color glass can be suitable for low-cost BIPV systems, and it has been proven that various colors can be implemented in a simple way to ensure the aesthetics of the building, while minimizing the reduction in solar power efficiency.

## 2. Experimental Section

### 2.1. Color Solution Formulation and Color Glass Fabrication Process

**Figure 1:**
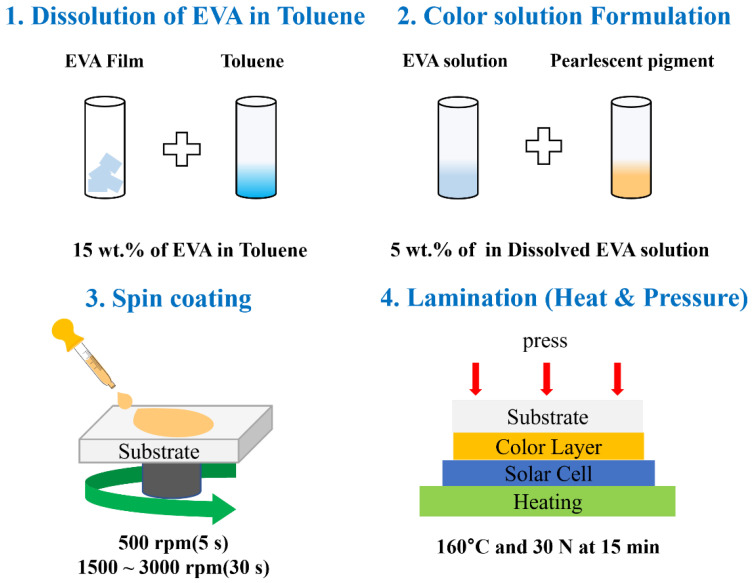
Solution formulation and color glass fabrication process.

This section describes the experimental preparation and color solution formulation methods for the fabrication of color glass. The size of the pearlescent pigment particles used in the experiment varies from 4 to 41 μm [27]. In addition, they are used for exterior paints and the interiors of automobiles, and various industrial paints, plastics, and cosmetics, so they are stable in the external environment. The pearlescent pigments (CQV, Korea) used in the experiment are AX-741 (Dazzling Red), AX-791K (Dazzling Green), A-781K (Splendor Blue), AX-901K (Dazzling Standard), AX-701K ((Dazzling Gold) and AX-761 (Dazzling Violet). The substrate used for the color solution coating is a normal glass substrate of 25 mm × 25 mm and mist glass. Normal glass and mist glass have a low iron content and high light transmittance compared to general glass, making them suitable as a substrate for BIPV [28]. The prepared glass substrate was ultrasonically cleaned with acetone for 15 min and then dried in an oven at 160 °C for 10 min. Conventionally, the thermal press method is also applied in the lamination processes of color glass and solar cells using EVA film, and since solar cell production is performed at a high temperature, the thermal press method performed for adhesion between color glass and the solar cell does not affect the characteristics of the solar cell. Figure 1 shows the experimental procedure for preparing color solutions. EVA was dissolved at a concentration of 15 wt.% using toluene, and 6 color solutions were prepared by mixing a 5 wt.% concentration of pearlescent pigment in the solution. In addition, the film adhesion was investigated for films prepared by dissolving EVA at concentrations of 10 wt.% and 20 wt.%, respectively. A spin-coating method was adopted for the film-coating process. The pre-cleaned glass substrate was pre-coated at 500 rpm for 5 s and then coated at 1500 rpm or 3000 rpm for 30 s to form a single-layer colored thin film, and it was left to cure in ambient conditions. In order to measure the transmittance and reflectance spectra of the prepared color glass, the optical properties of the color glass were measured in a wavelength band of 400~700 nm using an ultraviolet visible light near-infrared spectrophotometer (PerkinElmer, Waltham, MA, USA). The thickness of the color layer was measured according to the spin-coating speed by an α-step Dektak 150 (Veeko, Hongkong) profilometer.

### 2.2. Lamination and Solar Efficiency Measurement Method

**Figure 2:**
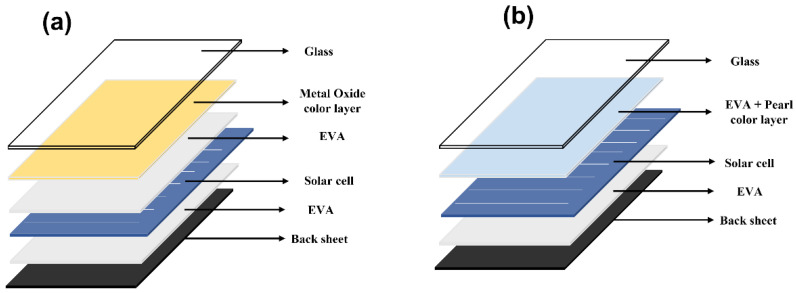
Schematic structure of color glass on solar cell. (**a**) Conventional structure. (**b**) Proposed structure.

After manufacturing the color glass, a heat press method was adopted, as shown in Figure 1. The setup was heated using a hot plate, and an object corresponding to about 30 N was placed on it. The solar cell and the color glass were laminated at a temperature of about 160 °C for 15 min. Pressure was applied during this process until no bubbles were seen in the film. The solar cell was manufactured based on crystalline Si and was provided by SHINSUNG E&G (Seongnam, Korea, Model: SH-2180S5P-D). Figure 2 shows the conventional color glass structure and the one proposed in this paper. Figure 2a is a representative multi-layer metal-oxide-based color glass, and the layer between the glass substrate and the solar panel is composed of a thin multi-layer colored film using metal oxide and an EVA film for lamination. It may take a long time and multiple steps for color glass to be fabricated and laminated on solar cells because the extra lamination step of employing EVA is involved. However, in the case of Figure 2b, dissolved EVA is used as a matrix for the color solution, so that it can be laminated directly to the solar panel in a heat press method. This architecture and procedure simplify the overall color glass structure and the fabrication steps. The proposed solution technique using dissolved EVA is suitable for industrial applications as it can effectively reduce time and cost compared to the conventional color glass manufacturing process. The color glass for testing with solar panels was manufactured by spin coating on a 3 × 8 cm^2^ of ordinary glass at a speed of 1000 rpm, and The solar power generation efficiency was measured using K201LAB50 (McScience, Suwon, Korea), and the voltage and current were predicted and displayed through the solar simulator.

## 3. Results and Discussion

### 3.1. Characterization of Thin Color Film and Color Glass

**Figure 3:**
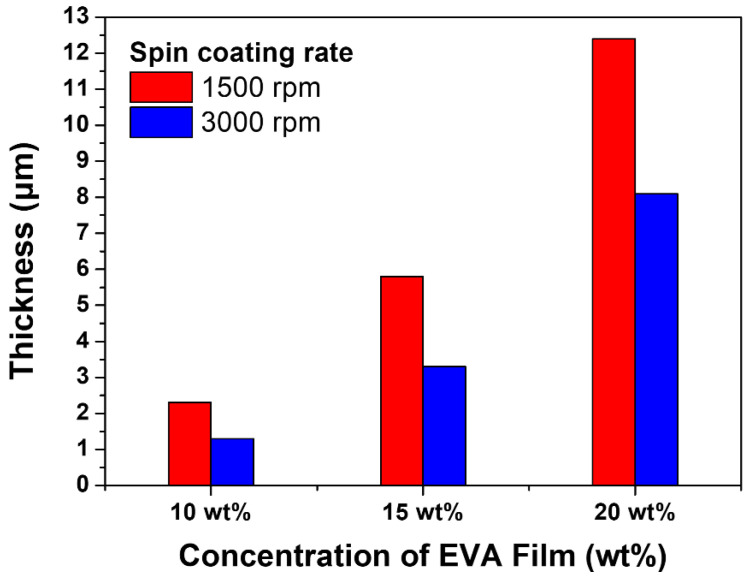
The thickness of the thin film at spin-coating speed of 1500 rpm (red line) and 3000 rpm (blue line) at 10, 15, and 20 wt.% EVA concentration.

Figure 3 shows the thickness of the thin film formed according to the concentration of the dissolved EVA and the spin coating speed. The thickness of the thin film is a factor that can affect the distribution of the pearlescent pigments and change the transmittance and reflectance spectra of the color glass. The color glass produced through the experiment exhibited a film thickness of 2.3~12.4 μm, depending on the concentration of EVA, when coated at a speed of 1500 rpm. There is a slight difference in thickness of 1.1 μm at 10 wt.%, 1.5 μm at 15 wt.%, and 3.1 μm at 20 wt.%, according to the spin-coating speed. However, depending on the concentration and spin-coating speed, the 1.3~2.3 μm thin film formed at a concentration of 10 wt.% is thin and the adhesive function due to the dissolution was lowered.

Table 1 shows that the thin film formed at a concentration of 15 wt.% showed stable adhesion to the solar panel. All EVAs used as solvents contained the same concentration of vinyl acetate (VA). However, it was expected that the concentration of VA would decrease due to dissolution and the adhesion function would decrease due to the thin film thickness. Although the adhesion to a solar cell is possible even at low concentrations of 10 wt.%, at least a concentration of 15 wt.% is required for secured lamination. However, as the concentration of EVA increases, the film naturally increases in thickness. As the amount of EVA increases, the concentration of the pearlescent pigment must also increase for natural color realization. However, if the concentration of the pearlescent pigment is increased, the transmittance of the color glass may be lowered, thereby reducing the solar power generation efficiency. Therefore, in consideration of high transmittance and stable adhesion, the solution concentration of EVA was selected as 15 wt.%.

**Figure 4:**
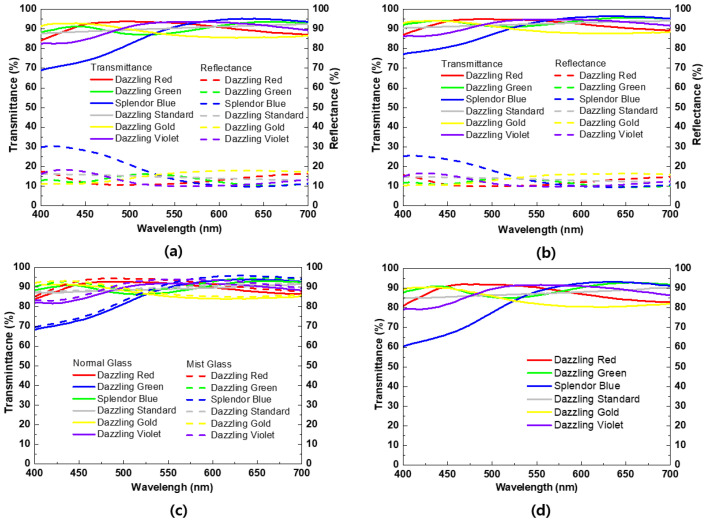
Transmittance and reflectance spectra of color glass spin coated at (**a**) 1500 rpm, and (**b**) 3000 rpm. Transmittance spectra of pearlescent pigments on normal and mist glass at spin-coating speed of (**c**) 1500 rpm and (**d**) 1000 rpm on 3 × 8 cm^2^ solar cell.

Figure 4 shows the transmittance and reflectance spectra of color glass. The color glass prepared by spin coating at 1500 rpm and 3000 rpm show high transmittance. All of the color glass produced by spin coating at 1500 rpm, as shown in Figure 4a, showed a maximum transmittance of over 90%. Dazzling Red showed a maximum reflectance of 16% at 675~700 nm and Dazzling Green showed a maximum reflectance of 490~540 nm. Splendor Blue, Dazzling Standard, and Dazzling Violet showed maximum reflectance of 30%, 16%, and 18%, respectively, at 400~440 nm. The three colors showed the highest reflectance in a common or nearby wavelength band, and Dazzling Gold showed a maximum reflectance of 17% at 605~650 nm. In the case of Splendor Blue, it showed a high reflectance spectrum, unlike other pearlescent pigments. This is because Splendor Blue consists of small particles of 3~30 μm, so the pigment particles are dense and the transmittance is low. In the case of coating at 3000 rpm, as shown in Figure 4b, the thickness of the color film decreased. In the case of Splendor Blue, the maximum reflectance decreased to 25% at 400 nm. In addition, the rest of the color glass showed a reduction in the maximum reflectance of 1~3%. The color solution mixed with dissolved EVA and pearlescent pigments shows a high transmittance of over 90% and this was expected to minimize the decrease in solar power generation efficiency. Figure 4c shows the transmission spectrum of color glass coated with each color solution prepared by spin coating at a speed of 1500 rpm on a normal glass and a mist glass substrate. The color glass produced using both glass types had a transmittance increase of 1~3% depending on the color. From the transmittance and reflectance spectra of the color glass, it is deduced that the transmittance increased and the reflectance decreased as the spin-coating speed increased. It is seen that, as the coating speed increases, the thickness of the thin film reduces and the distribution of the particles in the thin film is lowered. Figure 4d shows the transmittance of color glass prepared by spin coating 3 × 8 cm^2^ on ordinary glass at a speed of 1000 rpm to measure solar efficiency. Dazzling Red showed a maximum transmittance of 92% at 465~505 nm, Dazzling Green at 92% at 620~695 nm, Splendor Blue at 92% at 610~655 nm, Dazzling Standard at 90% at 675~700 nm, Dazzling Gold showed a maximum transmittance of 90% at 405~450 nm, and Dazzling Violet at 91% at 515~605 nm showed lower transmittance due to the increased thickness compared to the color glass manufactured at 1500 rpm and 3000 rpm. The spin-coating speed was selected as 1000 rpm because toluene is vaporized during coating, and as the glass substrate becomes larger, the pearlescent pigment cannot coat all areas, so the coating was performed at a 1000 rpm.

**Figure 5:**
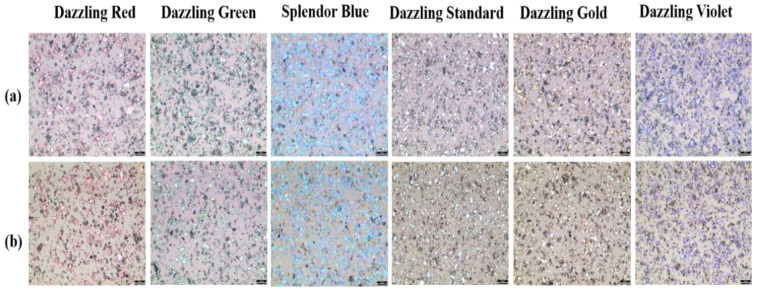
Optical microscope images of thin colored films spin-coated at (**a**) 1500 rpm and (**b**) 3000 rpm.

The particle distribution of the pearlescent pigment coated on the color glass was analyzed using an optical microscope. Figure 5 shows the distribution of pearlescent pigment particles distributed in the thin colored film. Figure 5a is a color glass manufactured with a spin-coating speed of 1500 rpm, and Figure 5b is a color glass manufactured by spin coating at 3000 rpm. Through the transmittance spectrum, it was confirmed that the transmittance increased by 2~5% when the spin-coating speed was increased. It can be seen from the optical microscope image that the amount of reflected light was small and the transmittance was increased as the distance between the particles increased.

**Figure 6:**
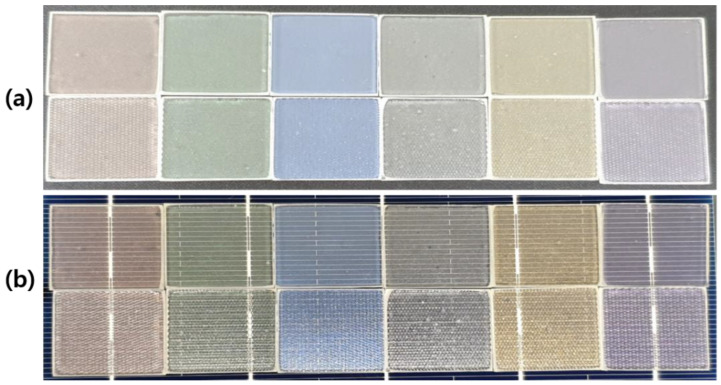
Actual photographs of color glass spin coated at 1500 rpm on (**a**) a black background and (**b**) on a solar cell. (First row: color glass on normal glass substrate, second row: color glass on mist glass substrate. From left to right: Dazzling Red, Dazzling Green, Splendor Blue, Dazzling Standard, Dazzling Gold, and Dazzling Violet).

Figure 6 shows the actual photographs of the manufactured color glass on the normal glass and the mist glass substrate. (The upper row is a normal glass substrate, and the lower row is color glass using a mist glass substrate). Figure 6a is color glass on a black background and Figure 6b shows color glass on a solar panel. Color glass manufactured using the solution process method is effective in hiding the dark monochromatic colors due to the uniform distribution of pearlescent pigments. The color solution in which EVA is dissolved can be laminated easily and simply by heat pressure method without additional use of EVA, as is seen in the case of metal-oxide-based color glass on solar panels. The proposed color glass manufacturing process is a factor that can effectively reduce the time and cost of the process, and the lamination process of color glass and solar panels can be performed quickly.

### 3.2. Stability of the Color Glass and the Effect on Solar Power Efficiency

**Figure 7:**
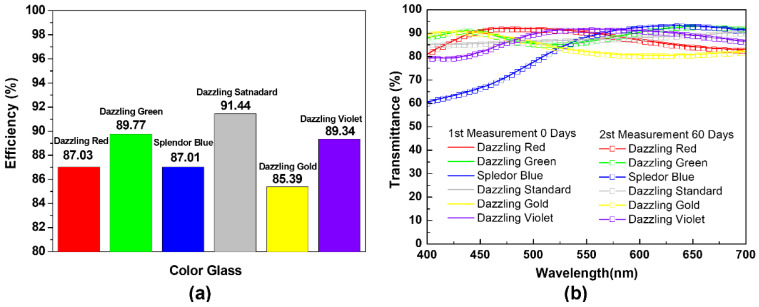
(**a**) Power generation efficiency evaluation of solar cells laminated with color glass. (**b**) Transmittance spectrum of color films measured after 60 days.

Figure 7 shows the solar power generation efficiency with color glass and the transmittance spectrum of color glass over time. Since color glass for BIPV is used as an exterior material for a building, it must be stable to the external environment and influence, and its properties must be maintained over time. The photovoltaic power generation efficiency was measured by making a 3 × 8 cm^2^ piece of color glass, storing it at room temperature, measuring the transmittance, and checking the change in optical properties over time after 60 days. Figure 7a is a graph measuring the power generation efficiency of a solar panel to which color glass is applied. Dazzling Red showed a maximum transmittance of 92% and power generation efficiency of 87%, Dazzling Green showed a maximum transmittance of 92% and power generation efficiency of 89%, Splendor Blue had a maximum transmittance of 92% and power generation efficiency of 87%, Dazzling Standard had a maximum transmittance of 90% and power generation efficiency of 91%, Dazzling Gold showed a maximum transmittance of 90% and a power generation efficiency of 85%, and Dazzling Violet showed a maximum transmittance of 91% and a power generation efficiency of 89%. Dazzling Standard, which has the highest power generation efficiency, has its transmittance over a large spectra range, unlike the transmission spectra of other colors, and it is shown that the power generation efficiency is high due to the uniform light transmittance over a large wavelength band. Next, the transmission spectra of Dazzling Green and Dazzling Violet, which exhibit similar power generation efficiencies, due to similar transmittance tendencies, but in alternating wavelength bands. In the case of the transmission spectrum of Splendor Blue, a sharp downward curve at 400~550 nm was seen, as compared to Dazzling Red. However, in the wavelength band above 550 nm, it showed higher transmittance than Dazzling Red, and as a result, it seemed to show a similar power generation efficiency. Similar to Dazzling Green and Dazzling Violet, Splendor Blue had a higher transmittance than Dazzling Red in the long wavelength band, so the power generation efficiency was similar. In the case of Dazzling Gold, the maximum transmittance was maintained only in the short wavelength band, and the transmittance decreased as it went to the longer wavelength band, so the power generation efficiency was lower than that of other color glass. In Figure 7b, the stability analysis after 60 days showed a slight change in transmittance of only 0.5%, showing that the optical characteristics of the color glass are stable over time. We have measured the angle-dependent transmittance of color glass (Figure S1). As the incident angle increased, the transmittance decreased significantly compared to the transmittance for vertical incident light. However, the color glass is attached parallel to the solar cell and must be considered at the module level where the color glass is applied. The angle-dependent transmittance of just the color glass is meaningless because color glass is almost always attached parallel to the solar cell modules. In future investigations, the issue of the efficiency change of the BIPV module with the angle of incidence must be considered. Therefore, it is demonstrated that color glass for BIPV systems, produced with dissolved EVA and a pearlescent pigment solution, achieves a power generation efficiency of 85~91%. These pieces of color glass are not intended to improve solar power generation efficiency, but they can minimize the decrease in solar power generation efficiency by securing the aesthetics of the building and maintaining a high transmittance.

## 4. Conclusions

In this paper, a color glass manufacturing process for BIPV using a color solution process method that uses pearlescent pigments to realize color was proposed. The matrix of the color solution was dissolved EVA. The color glass fabricated using the spin-coating method formed a thin single-layer colored film. The color glass had a thickness of 2.3~12.4 μm at 1500 rpm and 1.3~8.1 μm at 3000 rpm, depending on the color glass produced by the spin-coating method formed a single-layer colored film. In the thermocompression bonding method, the solar panel was pressed and adhered at 160 °C. for 15 min until there were no air bubbles in the film. Since EVA is used as a matrix for the color solution, it can be adhered to the solar panel using a heat press method without additional adhesive, effectively saving time and cost compared to the physical vapor deposition process. The color glass had a characteristic change of less than 0.5%, even with the passage of time, but the transmittance was maintained at 90% or more, and the distribution of pearlescent pigments was uniform, resulting in excellent esthetics. The solar power generation efficiency was up to 91% and is suitable as color glass for BIPV. The implementation of color glass for BIPV by the color solution process method proposed in this paper is suitable for use as an exterior material for buildings and is easy to manufacture, so it can be applied as an easy and simple low-cost process that can replace the physical vapor deposition process.

## Figures and Tables

**Table 1:** Lamination result according to EVA amount and concentration.

EVA Amount	5 wt.%	10 wt.%	15 wt.%	20 wt.%
EVA 1g	X	X	O	O
EVA 2g	X	O	O	O
EVA 3g	X	O	O	O

## Data Availability

The data presented in this study are available on request from the corresponding author.

## Supplementary Materials

The following supporting information can be downloaded at: https://www.mdpi.com/article/10.3390/ma15165570/s1, Figure S1: Angle-dependent transmission spectrum of a 3 × 8 cm^2^ solar cell spin-coated at 1000 rpm: (a) Dazzling Red, (b) Dazzling Green, (c) Splendor Blue, (d) Dazzling Standard, (e) Dazzling Gold and (f) Dazzling Violet.
