# Supplementary material for: Fabrication of Color Glass by Pearlescent Pigments and Dissolved EVA Film
Source: Materials (Basel). 2022 Aug 13;15(16):5570. doi: 10.3390/ma15165570 (PMC9416251; doi:10.3390/ma15165570)
Supplement: Supplementary file 1 [file materials-15-05570-s001.zip › materials-1836944-supplementary.pdf]

materials

Supplementary Materials

Fabrication of Color Glass by Pearlescent Pigments and Dissolved EVA Film

Seongmin Lim [1,†], Hyeon-Sik Ahn [1,†], Akpeko Gasonoo [2], Jae-Hyun Lee [3] and Yoonseuk Choi [1,*]

1 Department of Electronic Engineering, Hanbat National University, Daejeon 34158, Korea
2 Department of Chemistry, University of Calgary, 2500 University Drive N.W., Calgary, AB T2N 1N4, Canada
3 Department of Creative Convergence Engineering, Hanbat National University, Daejeon 34158, Korea
* Correspondence: ychoi@hanbat.ac.kr; Tel.: +82-42-821-1134
† These authors contributed equally to this work.

Citation: Lim, S.; Ahn, H.-S.; Gasonoo, A.; Lee, J.-H.; Choi, Y. Fabrication of Color Glass by Pearlescent Pigments and Dissolved EVA Film. *Materials* **2022**, *15*, 5570. https://doi.org/10.3390/ma15165570

Received: 12 July 2022
Accepted: 11 August 2022
Published: 13 August 2022

Publisher's Note: MDPI stays neutral with regard to jurisdictional claims in published maps and institutional affiliations.

Copyright: © 2022 by the authors. Licensee MDPI, Basel, Switzerland. This article is an open access article distributed under the terms and conditions of the Creative Commons Attribution (CC BY) license (https://creativecommons.org/licenses/by/4.0/).

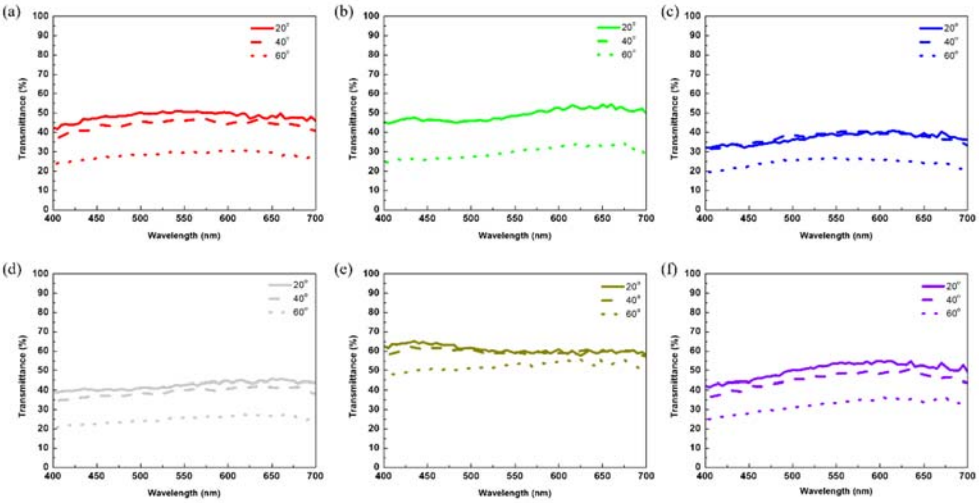

Figure S1. Angle-dependent transmission spectrum of a 3×8 cm^2 solar cell spin-coated at 1000 rpm (a) Dazzling Red (b) Dazzling Green (c) Splendor Blue (d) Dazzling Standard (e) Dazzling Gold (f) Dazzling Violet
